# Design, build and test of a targeted synthetic protein strategy

**DOI:** 10.3389/fbioe.2026.1798496

**Published:** 2026-07-29

**Authors:** Venkata V. B. Yallapragada, Arpit Shukla, Ciaran Devoy, Mark Tangney

**Affiliations:** 1 Cancer Research @UCC, University College Cork, Cork, Ireland; 2 Centre for Advanced Photonics and Process Analytics, Munster Technological University, Cork, Ireland; 3 APC Microbiome Ireland, University College Cork, Cork, Ireland; 4 School of Biotechnology & Bioengineering, Institute of Advanced Research, Gandhinagar, India

**Keywords:** antibody fragments single chain fv, computational protein design, in vivo bioluminescence imaging, synthetic biology (SB), targeted therapeutics

## Abstract

**Introduction:**

Incorporating minimal antibody regions into engineered proteins is an effective strategy for creating targeted therapeutics and diagnostics. However, a major barrier to clinical translation is the lack of efficient methods to track their performance *in vivo*. Bioluminescence imaging using targeted reporter proteins offers a promising solution to this challenge. The aim of this study was to develop a novel *in vivo* imaging strategy using computationally engineered targeted synthetic proteins and to establish a validated pipeline for their design, construction, and testing.

**Methods:**

Multi-part synthetic proteins were designed and screened using in silico tools. These constructs targeted either an example bacterial target (S. aureus ClfA) or a cancer biomarker (MUC1). The top candidates, featuring antibody fragments (ScFv) and a luciferase reporter, were subsequently produced and tested *in vitro* and in murine models.

**Results:**

Following computational design, select proteins were successfully produced and showed specific binding to their intended targets *in vitro*. Subsequent *in vivo* studies demonstrated that systemically administered proteins specifically accumulated at target cell locations. This was confirmed by localized bioluminescence that was dependent on both target cell number and protein quantity.

**Discussion:**

This study demonstrates the use of targeted reporter proteins for real-time *in vivo* imaging and validates an effective workflow integrating computational design with wet-lab experimentation. Ultimately, this establishes a target-specific reporter protein platform that can be adapted for sensitive *in vivo* imaging of diverse targets, from bacterial infections to tumors.

## Highlights

- An integrated *in silico* to *in vivo* platform for the rational design, screening, and validation of targeted synthetic proteins is established.
- The platform’s modularity is demonstrated by successfully engineering proteins against two clinically distinct targets: a bacterial virulence factor (*S. aureus* ClfA) and a human cancer antigen (MUC1).
- Systemically administered synthetic proteins demonstrate specific target accumulation and enable dose-responsive, non-invasive imaging in murine models of infection and cancer.
- The study provides a robust proof-of-concept for a versatile “design-build-test” strategy that can be rapidly adapted to new pathological targets.
- This work establishes a foundational framework for the future development of precision diagnostics and theranostic agents based on engineered protein scaffolds.

## Introduction

Protein engineering has become central to biomedical applications, particularly in the development of targeted imaging and therapeutic agents, leading to significant growth in protein-based imaging and therapeutics (Kontos et al., 2013; Walker et al., 2021). A well-established approach involves using antibodies as targeting agents; however, their large size can lead to challenges such as immunogenicity, slow clearance from the body, and poor tissue penetration (Khilji et al., 2023; Li et al., 2016). An effective alternative is the incorporation of minimal antibody regions into smaller, synthetic proteins. This strategy leverages the binding specificity of antibodies while improving pharmacokinetic properties and reducing the risk of an immune response. These engineered antibody fragments, such as single-chain variable fragments (ScFVs) and diabodies, can be assembled from the minimal binding regions of a full-sized antibody, offering a versatile and flexible design foundation with the potential for customized affinity, half-life, and valency (Bates and Power, 2019; Kothari et al., 2024).

Designing functional synthetic proteins requires the stable and energetically feasible assembly of multiple subparts, each with a unique function. These modular platforms, which can include elements like targeting domains, reporter proteins, and solubility enhancers, offered a powerful “design-build-test” strategy. The interchangeability of the targeting domain allowed for the rapid adaptation of the platform for various applications in diagnostics and therapy (Watson et al., 2023). However, the sheer number of possible variants for a multi-part protein made comprehensive wet lab testing both laborious and expensive. The use of *in silico* tools to model and screen these variants computationally provided a rational basis for design, helping to identify potential issues and select the most promising candidates for synthesis and further testing. This computational approach streamlines the development process, saving both time and resources (Walker et al., 2021).

This manuscript details the development and validation of a targeted synthetic protein platform, combining and condensing the findings from two distinct applications. The versatility of this modular, interchangeable system is demonstrated through comprehensive *in vitro* and *in vivo* data for two separate targeting domains. The first is aimed at the *Staphylococcus aureus* surface antigen Clumping factor A (ClfA), a key virulence factor in a clinically important pathogen. The second targets the tumour-associated MUC1 antigen, which is upregulated in various epithelial cancers and presented a promising strategy for cancer diagnosis and treatment (Grewal and Kurzrock, 2025). By presenting the complete workflow from computational design and *in silico* screening to wet-lab validation and preclinical *in vivo* imaging, this work provides a proof-of-concept for a robust and adaptable synthetic protein design strategy.

## Materials and methods

### *In silico* design and validation of synthetic proteins

The initial library of over 200 variants targeting either *S. aureus* Clumping factor A (ClfA) or human Mucin 1 (MUC1) were considered. Diversity was introduced by varying three design parameters: (1) Domain orientation (N-terminal vs. C-terminal fusion of the targeting domain relative to GLuc); (2) Linker composition and length (using (G_4_S)_n_ linkers where n = 1 to 4 to modulate domain flexibility); and (3) The inclusion or exclusion of solubility tags (Trx, SUMO). Diabody constructs were designed as single-chain Diabodies (scDb), where variable domains are connected by a short 5-amino acid linker to prevent intramolecular pairing. The binding domains were based on the variable heavy (VH) and light (VL) chains of anti-ClfA antibody MAb 12–9 and anti-MUC1 ScFv clone C595, respectively. Gaussia luciferase (GLuc) was incorporated as a luminescent reporter. A modular design approach was used, varying the arrangement of domains, the presence of solubility tags (e.g., Trx, SUMO), and the type and length of peptide linkers.

Candidate selection followed a defined exclusion funnel. First, structural stability was assessed; variants returning a C-score < −1.5 (a standard threshold for confident topology in I-TASSER indicating low fold confidence) or a positive Instability Index (>40, as defined by standard ProtParam algorithms) were discarded. Second, steric compatibility was evaluated visually in PyMOL; constructs where the linker length resulted in occlusion of the ScFv paratope by the luciferase domain were eliminated. Finally, the remaining candidates were ranked by predicted binding affinity (AutoDock Vina). Only variants demonstrating a theoretical ΔG lower than −10 kcal/mol and favourable solvent accessibility of the active site were advanced to synthesis. Three-dimensional structures were predicted using the I-Tasser protein modelling suite, and reliability was cross-referenced using the Rosetta modelling suite (Kaufmann et al., 2010; Yang and Zhang, 2015). The agreement between models was judged by the Root Mean Square Deviation (RMSD). The theoretical stability of the final models was validated using Ramachandran plots. Protein-protein docking simulations were performed using AutoDock Vina to predict binding affinity (ΔG) to the respective targets, ClfA and MUC1 (Trott and Olson, 2010). All 3D structures and docking conformations were visualized and analyzed using UCSF Chimera (Pettersen et al., 2004). Key parameters such as solvent accessibility of active sites, surface hydrophobicity, and structural integrity were assessed to screen and select the most promising candidates for wet-lab synthesis. The *in silico* pipeline used in this study is documented in the Function2Form framework by (Yallapragada et al., 2020).

## DNA design, cloning, and plasmid construction

The amino acid sequences of the finalized constructs were reverse-translated into DNA sequences using the EMBOSS Backtranseq tool. The resulting DNA sequences were codon-optimized for expression in Chinese Hamster Ovary (CHO) cells using the optimization tool on the IDT website. Final DNA gene blocks were synthesized by Twist Bioscience.

The synthesized gene fragments were cloned into the OG176 plasmid, which carries Kanamycin resistance, using Gibson Assembly (Gibson et al., 2009). The assembly reactions were transformed into competent *E. coli* BL21 cells, which were then plated on LB agar containing 50 μg/mL Kanamycin. Plasmids were extracted from overnight cultures using the Monarch Plasmid Miniprep Kit (New England Biolabs). Successful cloning and sequence integrity were confirmed in selected colonies by colony PCR using Q5 polymerase (NEB) and subsequent Sanger sequencing.

### Protein expression and production

CHO K1 cells were used for the production of all synthetic proteins. Cells were transfected with the expression plasmids using the Turbofect transfection reagent (Thermofisher) according to the manufacturer’s protocol. The supernatant containing the secreted synthetic proteins was collected at 24 and 48-h intervals post-transfection for subsequent assays.

### *In vitro* binding and functional assays

To ensure comparable protein loading in binding assays, the concentration of fusion protein in crude supernatants was normalized based on Gaussia luciferase activity (RLU). A standard curve using purified recombinant GLuc was used to estimate the mass equivalent of the fusion proteins ensuring that 1 µg equivalent of functional protein was applied in all comparative assays. To normalize protein input, the concentration of functional fusion protein in crude supernatants was estimated based on Gaussia luciferase activity (RLU) relative to a standard curve of purified recombinant GLuc. This normalization method operates on the assumption that the specific enzymatic activity of GLuc remains constant regardless of the attached ScFv, diabody, or solubility tag. While this assumption may introduce a systematic bias if specific activity varies between fusions, it provides a high-throughput proxy to ensure input normalization is based on properly folded reporter domains rather than total, potentially misfolded protein mass. All luminescence measurements were performed in triplicate using a Promega GloMax® 96 luminometer with Coelenterazine as the substrate. For the ClfA-targeted proteins, *S. aureus* TCH959 cells (10^8^ cells per sample) were blocked with 5% BSA for 2 h before incubation with the protein-containing supernatant. After incubation, cells were washed three times with PBS, resuspended, and assayed for bound luminescence.

For the MUC1-targeted proteins, binding was assessed using MCF7 (hMUC1-positive) and B16 (hMUC1-negative) cell lines. Cells at various concentrations were blocked with 5% BSA for 2 h, followed by incubation with the supernatant containing the test constructs. Cells were then washed three times with PBS before luminescence measurement. A subset of the *in vitro* experimental data reported here was originally presented in F2F-Bridge computational screening framework (Yallapragada et al., 2020); in that work, the data served to benchmark *in silico* construct selection. In the present study, these data are contextualised within a complete Design-Build-Test platform, where they serve as the *in vitro* selection step preceding *in vivo* validation.

### *In vivo* studies

All animal procedures were performed in accordance with Health Products Regulatory Authority (HPRA) ethical guidelines and were approved by the animal ethics committee at University College Cork. Studies were conducted using 6-8-week-old female BALB/c mice. For bacterial targeting studies, 10^7^ *S. aureus* cells were administered intramuscularly into the left quadriceps of anesthetized mice. For cancer targeting studies, 10^7^ MCF7 cells and 10^7^ B16 cells were injected subcutaneously on the left and right sides of the mouse, respectively. To assess immediate targeting specificity prior to the development of complex tumor stroma, systemic administration of synthetic proteins was performed within 1 h of cancer cell administration. Systemic administration of synthetic proteins (50–100 µL of supernatant) was performed via intravenous (IV) injection into the lateral tail vein. For imaging, 50 µL of Coelenterazine was injected at the target site 20–30 min after protein administration. Non-invasive bioluminescence imaging was conducted using the IVIS Lumina II Imaging system on mice kept under isoflurane-induced anaesthesia. Image analysis was performed using IVIS Living Image software.

## Results

### Rational design and in silico screening

**FIGURE 1:**
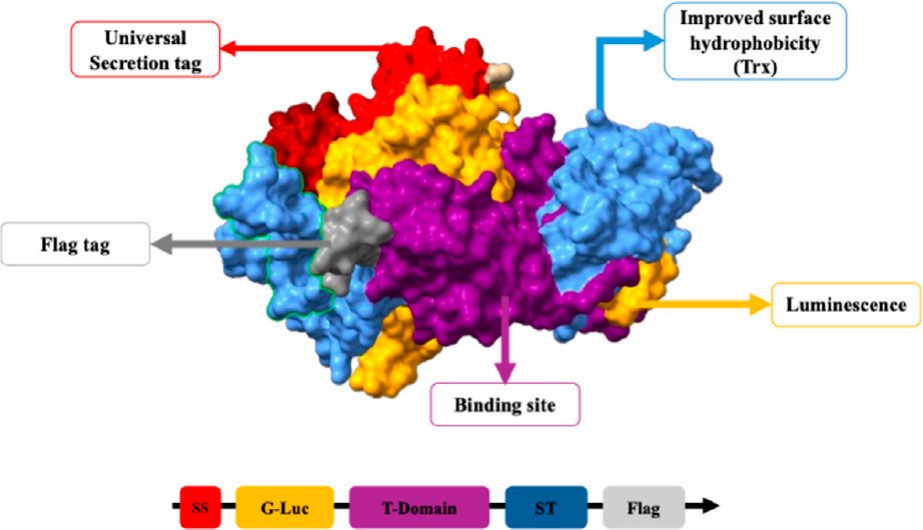
Modular Design of the Targeted Synthetic Protein Platform: A 3D structural model (top) and linear schematic (bottom) illustrate the modular architecture of the synthetic proteins. The platform includes a secretion signal (SS), a G-Luc reporter, an interchangeable targeting domain (T-Domain, magenta), a solubility tag (ST), and a Flag tag. The T-Domain, either an ScFv or diabody, is the variable component that directs specificity to targets such as ClfA or MUC1**.**

A comprehensive *in silico* screening funnel was employed to rationally design and select the most promising synthetic protein variants for wet-lab validation (Figure 1). The initial library was filtered based on three sequential criteria: (1) thermodynamic stability (Instability Index <40), (2) steric freedom of the binding domain (Linker length optimization), and (3) predicted binding energy (ΔG). Over 200 initial designs targeting either *S. aureus* ClfA or human MUC1 were considered and 50 computationally assessed. This process evaluated critical parameters including structural stability (RC score), folding confidence (C-score), predicted binding affinity (ΔG from docking), and the solvent accessibility of functional domains such as the paratope and secretion tags (Table 1). For example, protein modelling and visualization successfully identified and corrected design flaws, such as buried secretion tags that would likely result in non-functional proteins, by optimizing linker lengths to ensure proper domain exposure. This rational elimination of variants with a high probability of failure resulted in the selection of 10 final constructs targeting ClfA (4 ScFv, 4 diabody, and 2 controls) and 8 final constructs targeting MUC1 (4 ScFv, 2 diabody, and 2 controls) for synthesis and experimental testing.

**TABLE 1:** Key computational metrics for the final protein variants selected for experimental validation, grouped by their respective targets. Metrics include the C-score (confidence score from I-Tasser), TM-score (template modeling score), RC-score (Ramachandran plot score), solvent accessibility of the active site, free energy change from docking (ΔG), molecular size, and instability index. Molecular weight differences between M (ScFv) and MD (diabody) constructs reflect the duplication of the VH–VL domains in the single-chain diabody format, which approximately doubles the size of the targeting domain.

Construct Id	C-score (I-Tasser)	RC-score (%)	Hydrophobicity (%)	Active site accessibility (%)	Docking affinity (ΔG)	Size (kDa)	Instability index (%)
S1	0.057	78.1	45.88	61.1	−15.1	67.67	36.21
S2	0.114	86.2	47.11	64.88	−15.4	54.47	31.08
S3	0.071	82.8	46.66	57.77	−18.3	67.67	35.39
S4	0.147	86.5	47.11	59.55	−16.9	54.47	31.08
SD1	0.045	74.9	46.11	65.33	−13.6	99.03	38.42
SD3	0.182	80.2	46.88	65	−15.2	85.83	35.97
SD2	0.167	89.0	45.33	NA	NA	37.53	30.92
SD6	0.077	86.9	47.77	NA	NA	24.33	18.42
M1	0.04	84.3	44.787	59.888	−17.5	66.0	42.74
M2	0.17	88.4	45.777	60.0	−18.0	53.3	39.74
M3	0.08	83.5	44.888	62.222	−16.7	66.5	42.11
M4	0.22	86	45.8	61.55	−18.6	53.3	39
MD1	0.07	81.8	44.78	67.75	−16.3	82.9	47.75
MD3	0.0014	75.5	45.19	67.222	−13.4	83.46	46
MD2	0.16	89.0	45.333	NA	NA	37.53	30.92
MD6	0.077	86.9	47.777	NA	NA	24.33	18.42

### *In vitro* characterization of candidates

**FIGURE 2:**
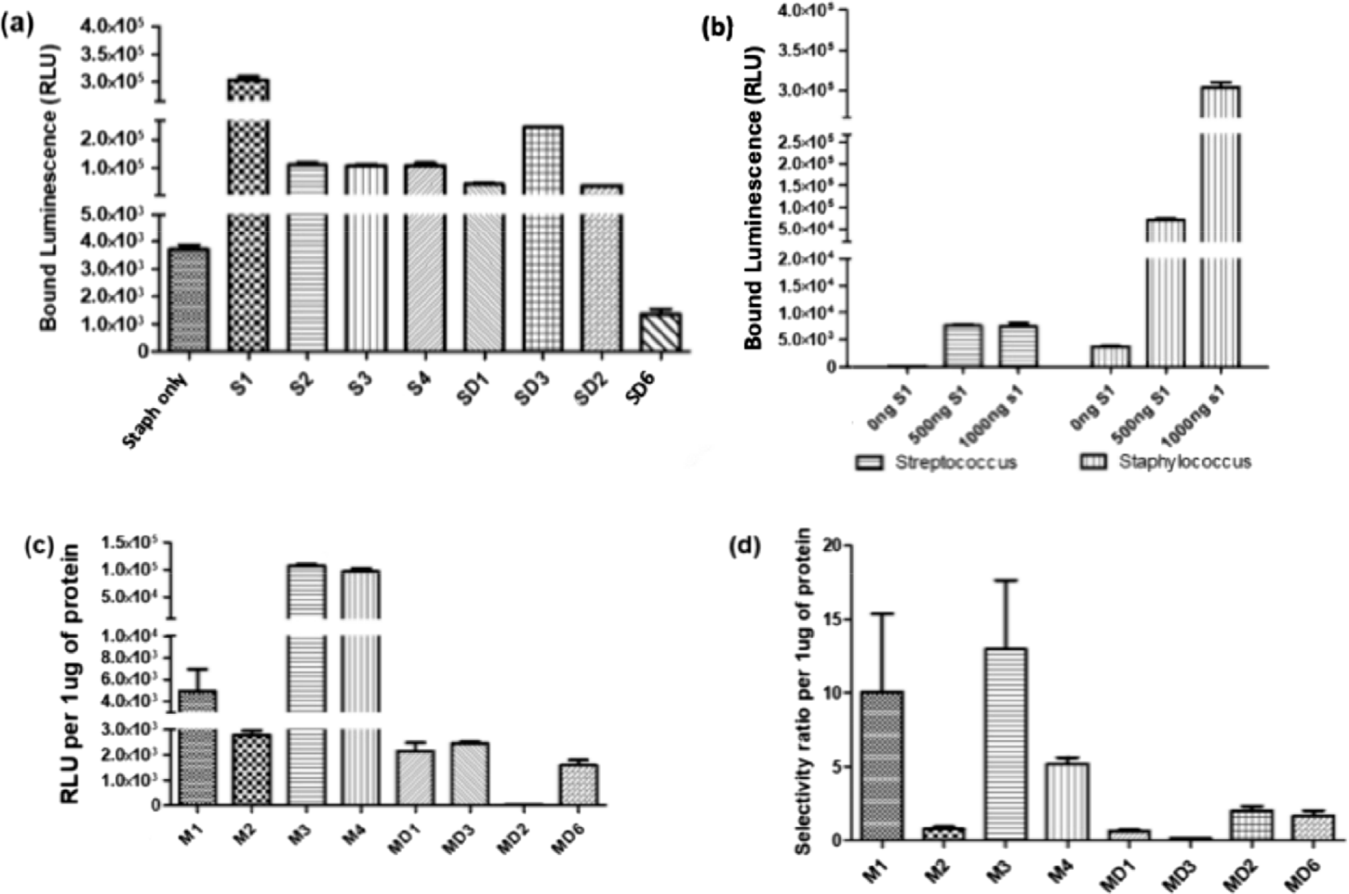
*In vitro* validation and selection of lead candidates. **(a)** *Anti-ClfA constructs bound luminescence*: Luminescence after binding to ClfA on *S. aureus*. 10^8^ *S. aureus* cells were incubated with 1 µg of each test construct for 1 h. Cells without treatment were used as a background. SD2 and SD6 were used as negative controls for binding. S1 (ScFv) emitted highest bound luminescence. ScFv construct S1 demonstrated a significantly higher raw binding signal to *S. aureus* compared to other variants. **(b)** Dose-dependent binding of S1 to *S. aureus*: 0, 500 ng and 1,000 ng of S1 was incubated with 10^8^ *S. aureus* or *Streptococcus agalactiae* cells. Bound luminescence was measured after three PBS washes. **(c)** *Bound luminescence of test variants to MUC1*: Luminescence after binding to MUC1 on MCF7 cells. 10^6^ MCF7 cells were incubated with 10 µL of each test construct for 1 h. Bound luminescence was normalised to protein quantity. **(d)** *Selectivity of synthetic proteins towards MUC1 positive cell lines* was calculated by calculating ratio between bound luminescence (per amount of protein added) from MCF7 cells and B16 cells. Based on this combination of high signal, specificity, and production, *S1* and *M1* were selected as lead candidates. Data are mean ± SEM (In all the constructs “M” refers to constructs targeting MUC1, “S” refers to constructs targeting ClfA. “MDx or SDx” are a diabody style design and “Mx” or “Sx” are ScFv style design). All experiments were performed in triplicate.

Following synthesis, all selected constructs were expressed in CHO cells, and their performance was evaluated *in vitro*. A subset of the *in vitro* experimental data presented here was previously reported in Yallapragada et al. (2020), where it served to benchmark the F2F *in silico* approach. Successful secretion and structural integrity of the fusion proteins were confirmed via luciferase activity assays. The detection of robust bioluminescence in the cell supernatant confirmed that the GLuc reporter domain was folded and active, serving as a functional proxy for the presence of the full-length fusion protein. For the ClfA-targeted constructs, luminescence assays on the cell supernatant confirmed that all variants were successfully secreted, with the control proteins lacking the targeting domain (SD2 and SD6) showing the high production levels (data not shown). In subsequent binding assays, the ScFv variant S1 demonstrated the highest luminescent signal upon binding to *S. aureus*, significantly outperforming all other constructs (Figure 2a). The specificity of S1 for *S. aureus* was further confirmed by dose-response experiments demonstrating selective binding over *Streptococcus agalactiae* (ClfA-negative)**.** Furthermore, the specific interaction with ClfA was validated by pre-blocking the target with human fibrinogen, which resulted in a concentration-dependent decrease in S1 binding of up to 68% (Figure 2b).

Similarly, the 8 constructs targeting MUC1 were evaluated. The M1 construct showed the highest level of protein production and yielded the highest raw luminescent signal in binding assays with MUC1-positive MCF7 cells. However, selecting the optimal candidate proved more complex; when luminescence was normalized to protein quantity, constructs M3 and M4 showed superior performance (Figure 2b). In selectivity assays comparing binding to MCF7 cells versus MUC1-negative B16 cells, M1 and M3 demonstrated the highest selectivity ratios (Figure 2d). Although constructs M3 and M4 demonstrated superior normalised binding performance per microgram of protein, M1 was selected as the lead candidate based on its highest absolute raw luminescent signal output combined with a high target selectivity ratio. For *in vivo* imaging applications, absolute signal intensity is a critical determinant of imaging sensitivity - insufficient total signal output will fail to produce a detectable bioluminescent image regardless of per-protein binding efficiency. M1 therefore represented the optimal combination of signal intensity and selectivity for progression to *in vivo* studies.

### *In vivo* imaging of infection and tumours

**FIGURE 3:**
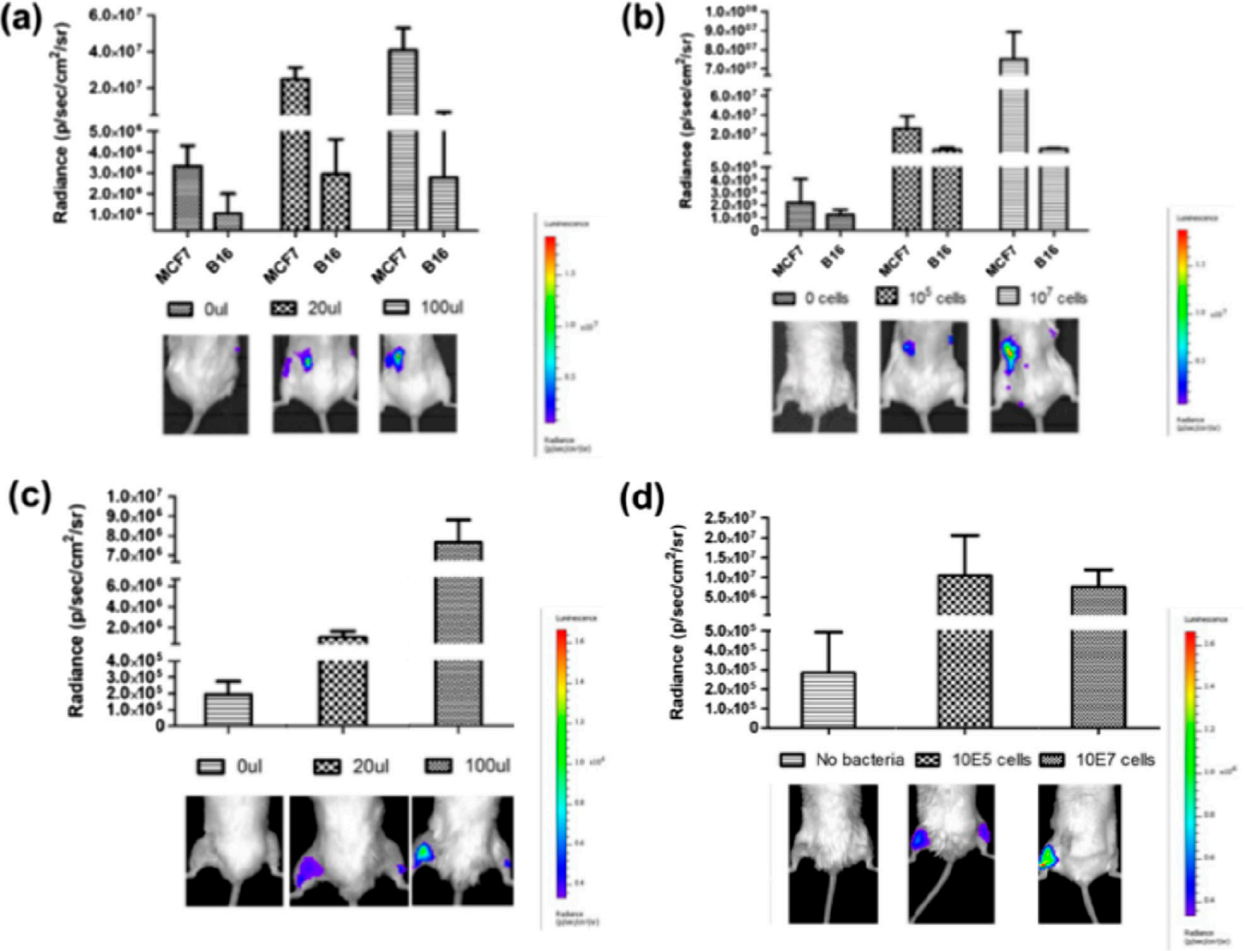
Lead candidates enable targeted and dose-responsive *in vivo* imaging. **(a)** *M1 dose response and target specificity*: 0 μL, 20 μL and 100 µL of M1 was administered systemically via tail vein to mice bearing both s. c. MUC1+ve (MCF7) and -ve (B16) cells. An increase in luminescence was observed with respect to the increase in synthetic protein concentration. Luminescence from left subcutaneous pocket, with MCF7 cells, produced a significantly (p 0.0087) higher signal than the right side, with B16 cells. **(b)** *In vivo target cell dose response*. A fixed dose of M1 was able to detect varying tumour burdens An increase in luminescence was observed in relation to the increase in number of MUC1+ve cells. Luminescence from left subcutaneous pocket, with MCF7 cells, produced a higher signal than the right side, with B16 cells (Test proteins were administered to mice within 1 h of cancer cell administration) **(c)** *S1 dose response:* 0 μL, 20 μL and 100 µL of S1 was administered systemically via tail vein. 10^7^ *S. aureus* cells were injected (IM) on the left quadriceps of mice. A dose-dependent signal was observed at the site of *intramuscular S. aureus* infection*.* **(d)** *Bacterial cell dose response:* 100 µL of S1 was administered systemically via tail vein. 0, 10^5^ and 10^7^ *S. aureus* cells were injected (IM) on the left quadriceps of mice. Representative bioluminescence images are shown for each condition. Data are mean radiance ±SEM (In all the constructs “M” refers to constructs targeting MUC1, “S” refers to constructs targeting ClfA. “MDx or SDx” are a diabody style design and “Mx” or “Sx” are ScFv style design). All experiments were performed in triplicate.

To validate the platform’s utility in a preclinical setting, the lead candidates M1 (anti-MUC1) and S1 (anti-ClfA) were assessed in murine models. Systemic (IV) administration of M1 into mice bearing both MUC1-positive (MCF7) and MUC1-negative (B16) subcutaneous tumours resulted in specific accumulation and a strong luminescent signal only at the site of the MCF7 tumour (Figure 3a). This signal was dose-dependent, increasing with higher concentrations of administered M1. Furthermore, the signal intensity correlated directly with the number of cancer cells present, demonstrating the ability to detect varying tumour burdens (Figure 3b).

To demonstrate the modularity and versatility of the design platform, the lead ClfA-targeted construct, S1, was also validated *in vivo*. Following systemic administration, S1 successfully localized to the site of an intramuscular *S. aureus* infection (Figure 3c). The resulting luminescent signal was again dose-dependent, with higher concentrations of S1 producing a brighter signal. The S1 construct was also able to detect different bacterial loads, confirming its potential for imaging infections *in vivo* (Figure 3d). Collectively, these results validate the entire design-build-test strategy, demonstrating its capability to produce targeted synthetic proteins that function specifically *in vitro* and enable dose-responsive imaging of distinct disease targets *in vivo*.

## Discussion

This study demonstrates the successful implementation of a comprehensive design-build-test pipeline for creating targeted synthetic proteins, culminating in a versatile and modular platform for *in vivo* imaging. By integrating rational *in silico* design with empirical *in vitro* and *in vivo* validation, a robust proof-of-concept was established, validating the platform against two clinically distinct and highly relevant targets: the bacterial virulence factor ClfA on *S. aureus* and the oncogenic MUC1 antigen on cancer cells. The key achievement of this work is not the creation of a single-purpose agent, but the validation of a systematic and adaptable workflow capable of generating bespoke imaging probes for diverse pathologies (Kontos et al., 2013).

A critical finding of this study is that no single *in silico* parameter reliably predicts *in vitro* binding performance. Construct S3, for example, exhibited superior computational metrics compared to lead candidate S1 across multiple parameters yet failed to bind effectively *in vitro*. This dissociation between *in silico* scores and wet lab performance underscores the rationale for the multi-parameter screening funnel and subsequent empirical validation, and argues that computational tools are most powerful as filters to eliminate unsuitable candidates rather than as definitive predictors of the best performer.

**FIGURE 4:**
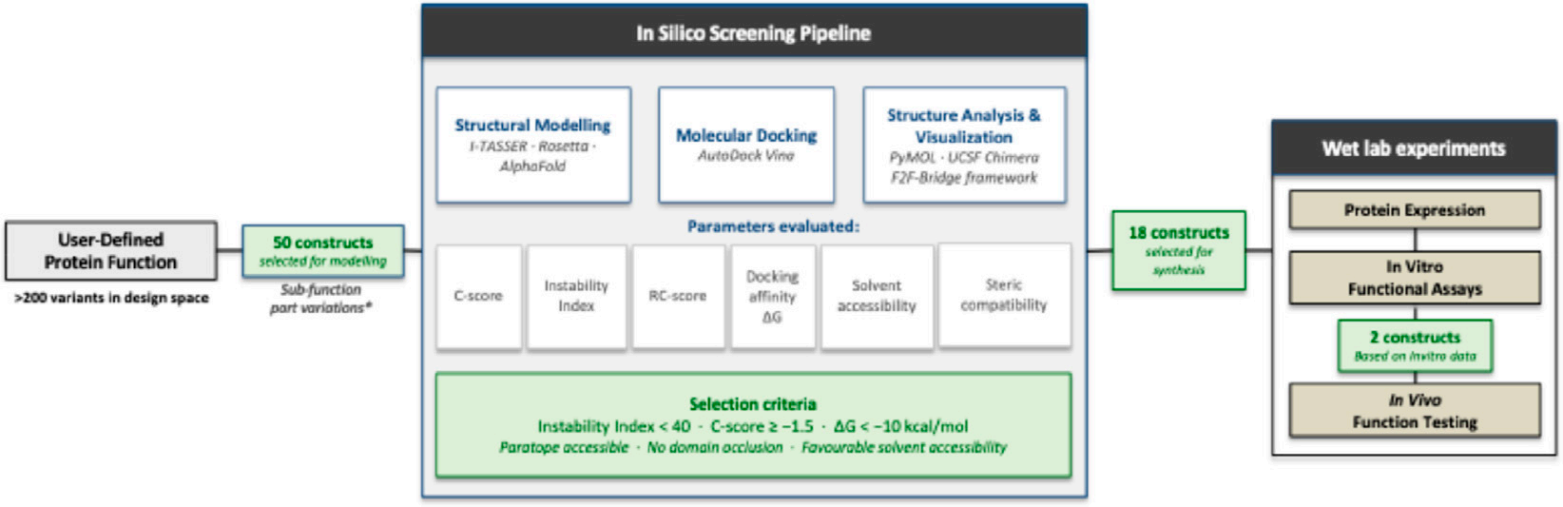
Design-build-test workflow for targeted synthetic protein development. Sub-function parts were permuted to generate >200 candidate constructs, from which 50 representative constructs were selected for computational screening. 18 constructs satisfying all *in silico* selection criteria were advanced to experimental validation, with two lead constructs identified for *in vivo* testing.

Our approach (See Figure 4) is firmly rooted in the principles of modern synthetic biology and computational protein engineering, which have become indispensable for accelerating the development of novel biologics. While traditional discovery methods rely on large-scale, laborious experimental screening, the initial *in silico* funnelling process allowed for the rapid assessment of hundreds of designs, efficiently eliminating variants with predicted structural flaws or poor target engagement. This strategy is consistent with a paradigm shift in biologics design, where computational tools are increasingly used to de-risk and prioritize candidates before committing to costly and time-consuming wet-lab synthesis (Devoy et al., 2024; Sadybekov and Katritch, 2023). While the workflow relied on established modelling algorithms, the next frontier lies in integrating deep learning-based tools like AlphaFold, which have revolutionized protein structure prediction and could further enhance the accuracy of *de novo* design and affinity prediction in future iterations of this platform (Jumper et al., 2021; Marcu et al., 2022).

A critical finding of this study was that predicted binding affinity (ΔG) alone did not guarantee *in vitro* performance. For example, while construct S3 exhibited a superior docking score (−18.3 kcal/mol) compared to the lead candidate S1 (−15.1 kcal/mol), it failed to bind effectively *in vitro*. Retrospective structural analysis suggests this discrepancy is primarily driven by domain orientation. While the static docking model for S3 showed a thermodynamically favourable interaction, the geometry of the linker likely positioned the bulky GLuc reporter such that it caused a steric clash with the bacterial cell surface upon target approach. Conversely, the optimized geometry in S1 orientated the reporter away from the ScFv paratope, ensuring unobstructed access to the ClfA antigen. This highlights a limitation of static protein-protein docking models and demonstrates that future iterations of this predictive pipeline must incorporate dynamic steric modelling to assess full-construct orientation relative to the target surface.

The modular nature of the proposed platform, which allows for the interchangeable use of targeting domains, is a significant strength. Antibody fragments (ScFvs and diabodies) that retain the high specificity of monoclonal antibodies but offer superior pharmacokinetics for imaging, such as faster clearance and potentially improved tissue distribution compared to full-length antibodies were successfully deployed in the present study (Holliger and Hudson, 2005; Lu et al., 2025; Weisser and Hall, 2009). This “plug-and-play” capability positions the platform as a powerful tool for rapidly addressing new targets. For instance, the successful imaging of *S. aureus* infections highlights a critical application. Current clinical standards for diagnosing deep-seated infections often rely on non-specific imaging modalities or complex radiopharmaceutical-based methods like ^18^F-FDG-PET, which can struggle to differentiate infection from sterile inflammation (Ordonez and Jain, 2018). A bioluminescence-based approach provides a highly sensitive, non-radioactive alternative for preclinical research, enabling high-throughput evaluation of anti-infective therapies and offering a pathway toward novel diagnostics that directly target the pathogen (Kunjachan et al., 2015; Maruthi and Kalangi, 2021). While the platform successfully targeted both bacterial and tumour antigens, certain limitations exist. The current design relies on the accessibility of extracellular epitopes; targets buried within deep tissue clefts would require alternative delivery strategies. Furthermore, the addition of the luciferase reporter (19 kDa) may introduce steric hindrance for smaller targets. Future iterations could employ smaller reporters, such as NanoLuc, to mitigate steric clashes.

Methodologically, quantifying crude fusion proteins via reporter activity relies on the assumption of uniform specific activity across all variants. Alterations in GLuc enzymatic efficiency due to different fusion domains could introduce systematic bias in concentration normalization. However, the magnitude of the signal differences observed between the lead candidates and non-functional variants strongly supports specific target engagement over artifacts of enzymatic bias. Additionally, the *in vivo* cancer targeting was evaluated using an early-stage localization model (co-injection/immediate administration) to establish foundational targeting specificity. Future studies will need to evaluate these agents in established, vascularized solid tumours to assess tissue penetration through complex tumour stroma.

Similarly, the specific targeting of the MUC1 antigen demonstrates the platform’s potential in oncology. MUC1 is a prime target for various therapeutic strategies, including antibody-drug conjugates (ADCs), bispecific antibodies, and CAR-T cell therapies, due to its overexpression and aberrant glycosylation in numerous cancers (Grewal and Kurzrock, 2025; Long et al., 2023; Nath and Mukherjee, 2014). A significant challenge in the clinical application of these therapies is patient stratification and monitoring therapeutic response. The imaging agents developed through the platform could function as non-invasive companion diagnostics, enabling the visualization of MUC1 expression to select patients most likely to benefit from MUC1-targeted treatments or to assess target engagement and tumour response dynamically throughout a treatment course (Chen et al., 2024).

Looking forward, the validated platform serves as an ideal foundation for developing theranostic agents. By replacing the luciferase reporter with a therapeutic payload such as a cytotoxic drug for an ADC, a photosensitizer for photodynamic therapy, or a chelator for targeted radionuclide therapy; this framework could be used to create agents that combine diagnosis and treatment (Kelkar and Reineke, 2011; Woong Yoo et al., 2022). Before clinical translation, however, a thorough investigation of the immunogenicity, long-term toxicity, and detailed pharmacokinetics of these synthetic proteins is essential. *In silico* deimmunization algorithms could be integrated into the design pipeline to predict and eliminate potential T-cell epitopes, thereby engineering constructs with a lower risk of eliciting an adverse immune response (Mattei et al., 2022; Mattei et al., 2024).

In conclusion, this work validates a powerful and versatile platform for the rational design of targeted synthetic proteins. By successfully generating and validating imaging agents for both bacterial infection and cancer, the modularity and broad applicability of the presented design-build-test strategy is demonstrated. This research lays the groundwork for the future development of a new class of precision diagnostic and theranostic agents, with the potential to accelerate preclinical research and ultimately address unmet needs in the management of a wide range of human diseases.

## Conclusion

In this study, a modular platform was successfully developed and validated for the rational design and application of targeted synthetic proteins. By integrating computationally informed design with rigorous experimental validation, specific, high-affinity imaging agents were generated against two disparate and clinically significant targets: the bacterial antigen ClfA and the oncogenic marker MUC1. The successful systemic administration of the lead candidates followed by targeted localization and dose-responsive bioluminescent imaging in murine models of infection and cancer provides a definitive proof-of-concept for the entire design-build-test workflow. This work lays the foundation for the rapid development of a new class of precision biologics, demonstrating a versatile and adaptable strategy with broad potential for application in preclinical imaging, diagnostics, and the future creation of targeted theranostics for a multitude of human diseases.

## Data Availability

The datasets presented in this study can be found in online repositories. The names of the repository/repositories and accession number(s) can be found in the article/Supplementary Material.
