# Supplementary material for: Design, build and test of a targeted synthetic protein strategy
Source: Front Bioeng Biotechnol. 2026 Jul 29;14:1798496. doi: 10.3389/fbioe.2026.1798496 (PMC13463047; doi:10.3389/fbioe.2026.1798496)
Supplement: Supplementary file 1 [file DataSheet1.pdf]

ST1 - Design Rationale for *In Silico* screening

Part	Considered for design	Decision making details & Triage Rationale	Proceeding for *in silico* screening
**Secretion Signal Peptide**	GLuc SP, Kappa light chain SS	**GLuc SP (Eukaryotic):** Works for both bacterial and mammalian cell secretion. Preferred for greater reliability and efficiency.	**GLuc SP** is chosen (n=1).
**Linkers**	$(GGGGS)_3$, $(Gly)_8$, $(EAAAK)_3$, Di-Sulfide linkers, PAPAP	**$(GGGGS)_n$ (Flexible):** Rich in hydrophilic amino acids; allows domain interaction due to flexibility, increasing stability and folding. **$(EAAAK)_n$ (Rigid):** Forms alpha-helix structures; keeps distance between domains to maintain independent functions. Separates domains more effectively than flexible linkers.	**n=2 configurations:** Only flexible linkers $(GGGGS)_n$. Alternating flexible and rigid linkers between variable chains and ScFv fragments *(Note: Diabody requires a combination to keep chains separate but allow interaction).*
**Antibody Fragment**	VH fragment, ScFv, Diabody, Minibody, Antibody	Smaller fragments have faster blood and renal clearance.	**ScFv and Diabody** versions were modelled. The test variants differed in the order of arrangements.
**Imaging Element**	Gaussia Luciferase, Renilla Luciferase, GFP, UCNPs	**Gaussia Luciferase:** Chosen for its small size and because it is naturally secreted out of cells.	**GLuc** is chosen (n=1).
**Solubility Tag**	MBP, GST, Trx, NusA, SUMO	**Trx:** Small size, highly soluble, heat stable. Avoids inclusion body formation; highly suitable for *E. coli*. **MBP:** Huge size; used as a design control.	Models with **Trx** and **MBP** (n=2).
**Detection / Purification Tag**	Poly-His Tag, Flag, c-Myc	**Flag:** Short, hydrophilic, and readily available commercial assays. **His:** Most common, short, allows denaturing purification, but not preferred for antibody detection.	Both **Flag and Poly-His** considered for computational modelling (n=2).

1. Chen, X., J.L. Zaro, and W.C. Shen, *Fusion protein linkers: property, design and functionality.* Adv Drug Deliv Rev, 2013. **65**(10): p. 1357-69.
2. Argos, P., *An investigation of oligopeptides linking domains in protein tertiary structures and possible candidates for general gene fusion.* J Mol Biol, 1990. **211**(4): p. 943-58.
3. Huston, J.S., et al., *Protein engineering of antibody binding sites: recovery of specific activity in an anti-digoxin single-chain Fv analogue produced in Escherichia coli.* Proc Natl Acad Sci U S A, 1988. **85**(16): p. 5879-83.
4. Trinh, R., et al., *Optimization of codon pair use within the (GGGGS)3 linker sequence results in enhanced protein expression.* Mol Immunol, 2004. **40**(10): p. 717-22.
5. Alfthan, K., et al., *Properties of a single-chain antibody containing different linker peptides.* Protein Eng, 1995. **8**(7): p. 725-31.
6. Arai, R., et al., *Design of the linkers which effectively separate domains of a bifunctional fusion protein.* Protein Eng, 2001. **14**(8): p. 529-32.
7. Hagemeyer, C.E., et al., *Single-chain antibodies as diagnostic tools and therapeutic agents.* Thromb Haemost, 2009. **101**(6): p. 1012-9.
8. Costa, S., et al., *Fusion tags for protein solubility, purification and immunogenicity in Escherichia coli: the novel Fh8 system.* Front Microbiol, 2014. **5**: p. 63.
9. Rosano, G.L. and E.A. Ceccarelli, *Recombinant protein expression in Escherichia coli: advances and challenges.* Front Microbiol, 2014. **5**: p. 172.
10. Fox, J.D., R.B. Kapust, and D.S. Waugh, *Single amino acid substitutions on the surface of Escherichia coli maltose-binding protein can have a profound impact on the solubility of fusion proteins.* Protein Sci, 2001. **10**(3): p. 622-30.
11. Kapust, R.B. and D.S. Waugh, *Escherichia coli maltose-binding protein is uncommonly effective at promoting the solubility of polypeptides to which it is fused.* Protein Sci, 1999. **8**(8): p. 1668-74.
12. Shih, Y.P., et al., *High-throughput screening of soluble recombinant proteins.* Protein Sci, 2002. **11**(7): p. 1714-9.
13. Kohl, T., et al., *Automated production of recombinant human proteins as resource for proteome research.* Proteome Sci, 2008. **6**: p. 4.
14. LaVallie, E.R., et al., *Thioredoxin as a fusion partner for production of soluble recombinant proteins in Escherichia coli.* Methods Enzymol, 2000. **326**: p. 322-40.
15. Marblestone, J.G., et al., *Comparison of SUMO fusion technology with traditional gene fusion systems: enhanced expression and solubility with SUMO.* Protein Sci, 2006. **15**(1): p. 182-9.
16. Panavas, T., C. Sanders, and T.R. Butt, *SUMO fusion technology for enhanced protein production in prokaryotic and eukaryotic expression systems.* Methods Mol Biol, 2009. **497**: p. 303-17.
17. Hammarstrom, M., et al., *Rapid screening for improved solubility of small human proteins produced as fusion proteins in Escherichia coli.* Protein Sci, 2002. **11**(2): p. 313-21.
18. Hammarstrom, M., et al., *Effect of N-terminal solubility enhancing fusion proteins on yield of purified target protein.* J Struct Funct Genomics, 2006. **7**(1): p. 1-14.
19. Smith, D.B., *Generating fusions to glutathione S-transferase for protein studies.* Methods Enzymol, 2000. **326**: p. 254-70.
20. Terpe, K., *Overview of tag protein fusions: from molecular and biochemical fundamentals to commercial systems.* Appl Microbiol Biotechnol, 2003.

60(5): p. 523-33.
21. Lichty, J.J., et al., *Comparison of affinity tags for protein purification.* Protein Expr Purif, 2005. **41**(1): p. 98-105.
22. Kimple, M.E., A.L. Brill, and R.L. Pasker, *Overview of affinity tags for protein purification.* Curr Protoc Protein Sci, 2013. **73**: p. Unit 9 9.
23. Bornhorst, J.A. and J.J. Falke, *Purification of proteins using polyhistidine affinity tags.* Methods Enzymol, 2000. **326**: p. 245-54.

Supplementary Table ST2: Amino Acid Sequences of Key Modular Components

Construct Component	Amino Acid Sequence
GLuc Signal Sequence	MGVKVLFALICIAVA
Gaussia Luciferase (GLuc)	EAKPTENNEDFNIVAVASNFATTDLDADRGKLPGKKLPLEVLKEMEANARKAGCTRGCLICLSHIKCTPKMKKFIPGRCHTYEGDKESAQGGIGEAIVDIPEIPGFKDLEPMEQFIAQVDLCVDCTTGCLKGLANVQCSDLLKKWLPQRCATFASKIQGQVDKIKGAGGD
Anti-MUC1 ScFv *(Based on C595 variable domain)*	QVQLQESGGGLVQPGGSLKLSCAASGFTFSSYGMSWVRQTPDKRLELVATINSNGGSTYYPDSVKGRFTISRDNAKNTLYLQMSSLKSEDTAMYYCARDRDGYDEGFDYWGPGTTVTVSSGGGGSDIELTQSPSIMSASPGEKVTMTCSASSSVSYMHWYQQKSGTSPKRWIYDTSKLASGVPARFSGSGSGTSYSLTISSMEAEDAATYYCQQWSSNPPTFGGRTKLELK
Anti-ClfA ScFv *(Based on MAb 12-9)*	QVQLKESGPGLVAPSQSLSITCAISGFSLSRYSVHWVRQPPGKGLEWLGMIWGGGNTDYNSALKSRLSISKDNSKSQVFLKMNSLQTDDTAMYYCARKGEFYYGYDGFVYWGQGTLVTVSAGGGSNIMMTQSPSSLAVSAGEKVTMSCKSSQSVLYSSNQKNYLAWYQQKPGQSPKLLIYWASTRESGVPDRFTGSGSGTDFTLTISSVQAEDLAVYYCHQYLSSYTFGGGTKLEIK
Trx Tag	MSDSEVNQEAKPEVKPEVKPETHINLKVSDGSSEIFFKIKKTTPLRRLMEAFAKRQGKEMDSLRFLYDGIRIQADQTPEDLDMEDNDIIEAHREQIGGAT
Flag Tag	DYKDDDDK

The anti-MUC1 ScFv was based on the C595 variable domain sequences (Denton et al., 1997)

Denton G, Sekowski M, Spencer DI, Hughes OD, Murray A, Denley H, Tendler SJ, Price MR. (1997). Production and characterization of a recombinant anti-MUC1 scFv reactive with human carcinomas. British Journal of Cancer, 76(5), 614–621. https://doi.org/10.1038/bjc.1997.434

The anti-ClfA ScFv was derived from the variable domain sequences of the murine monoclonal antibody MAb 12-9 (Hall et al., 2003)

Hall AE, Domanski PJ, Patel PR, Vernachio JH, Syribeys PJ, Gorovits EL, Johnson MA, Ross JM, Hutchins JT, Patti JM. (2003). Characterization of a protective monoclonal antibody recognizing Staphylococcus aureus MSCRAMM protein clumping factor A. Infection and Immunity, 71(12), 6864–6870. https://doi.org/10.1128/IAI.71.12.6864-6870.2003
